# The Influenza of 1918-1919

**Published:** 1922-02

**Authors:** A. T. Nankivell

**Affiliations:** (Medical Officer of Health, Hornsey)


### NOTABLE EPIDEMICS.

### IV.—THE INFLUENZA OF 1918-1919.

By A. T. NANKIVELL, M.D., D.P.H. (Medical Officer of Health, Hornsey).

THE outbreak of influenza which visited the world a few years ago caused in a few months more deaths than were due to the Great War. In England alone more than 150,000 persons perished from the disease. Had an epidemic of such magnitude visited our country in times of peace there would have been universal dismay and panic ; as it was, fully occupied as we were in winning a great war, the epidemic passed us by and took from us its toll of life without especially stirring the popular imagination.

The epidemic of influenza of which I write did not come unannounced. In the autumn and winter of 1917 numbers of cases of so-called "purulent bronchitis" were observed among the troops, both at home and abroad, and also among the civil population ; and in the late spring and early summer of 1918 there were vast numbers of cases of fever which lasted for a day or two and gave rise to little or no mortality. The fatal type of influenza began in October, 1918, and was characterised especially by its pulmonary complications that lead rapidly to death. After ravaging this country for two months, the epidemic died down—only to reappear in the February of 1919, when again it caused a larger mortality until the coming of the spring. Influenza returned again in February, 1920, but did not cause any great mortality, and it was recognised also in the early spring of 1921, when there were a large number of cases with very few deaths. Thus the epidemic of influenza which reached its height in the winter 1918-1919 was preceded by mild outbreaks and followed by mild outbreaks.

The symptoms of this disease are known by most of us ; and have been admirably described in many of the medical journals of the last few years. The onset was sudden, there was general pain and discomfort, and pain behind the eyes was a frequent complaint of the sufferer. Sometimes the whole face seemed to be acutely painful. Sore throat and nose bleeding were common, there was a rise of temperature, and a rise in the rate of respiration. The disease was accompanied by a feeling of profound lassitude and prostration. The sick person wanted to lie down and to be left alone A few rapidly fatal cases were marked by hæmorrhages into the skin, and in fatal cases bleeding into the muscles was not uncommon. Lung complications, which certainly were not pneumonias, became evident on the third or fourth day of illness. These complications often proved to be fatal during the course of a day or two. For those influenza lung conditions several names have been found, such as pulmonitis and pneumonitis ; but the name, after all, is not of much consequence. The lungs of the patients who died showed acute inflammation, and appeared as if the sufferer had been poisoned with some irritant gas, and had afterwards been drowned. It was rare to find pneumonia or broncho-pneumonia in any fatal case of influenza—but the hæmorrhaegic œdema, the "drowned lung," was of common occurrence. This complication, which caused almost all the influenza mortality, is readily understood when we remember that the poison of influenza (whatever it may be) is a nerve poison ; the respiratory nerves of these severe cases were paralysed or partly paralysed, and the patients were unable to cough up the blood and fluid in their lungs—hence their "drowned" appearance.

The influenza of 1918-1919 was an acutely infectious disease, and was spread from person to person. The incubation period was short—a few hours to three days ; and the infection was probably carried through the air ("droplet" infection). Certain persons, although exposed frequently to the risk of infection, seemed to be immune to the disease. Children and old people were not commonly attacked, and the burden of the disease fell upon the young adults. The mortality was greatest between the ages of twenty and forty, in distinction to the influenza of 1890, which attacked the older people. No cure was discovered for the lung complications, and no certain methods of preventing the disease are known. The use of gauze masks is not practical, nor is the prevention of overcrowding in railways, trams, streets and public meeting places. It seems likely that the prophylactic use of a mixed vaccine was effective in preventing some of the lung complications, but there is no evidence to show that such a vaccine reduced the incidence of influenza.

What caused this giant epidemic to arise after its long sleep since 1890 ? There are several theories regarding this great outbreak of influenza, and it is difficult to say where lies the right explanation.

It is held by some that the disease arose *de novo*, that it was a new thing caused by an "influence" from the stars, caused by the wickedness of men, or caused by our flying in aeroplanes in the face of Providence, or wearing soft collars or listening to jazz bands. Before accepting this theory of influenza we will consider whether some other is not more credible.

The overcrowding of persons, especially in times of stress and dangers, has given rise to epidemics in former years. That most famous town of Caffa, in the Crimea, was packed full of Italian merchants who had escaped thither from Tana on the Don, where they had been besieged by the Tartars. The Tartars followed the merchants to Caffa, and besieged them there for nearly three years. Bubonic plague broke out both within the city and outside it. De Mussis, who, perhaps, may have been one of the interned merchants in Caffa, relates how the Tartars by means of their engines of war threw their infected dead over the walls and into the city. No doubt the Italians thought of suitable reprisals. The result of the plague was, however, that the siege was raised, and the Italian merchants departed home, taking their fleas with them. They landed at Genoa, and two days later plague broke out in that town. This was the experience of the fourteenth century, and we can

**Notable Epidemics—(*cont.*).**

imagine something parallel having taken place in 1917 and onwards; for surely the civilised world was never so overcrowded as it was in these war years. Vast bodies of men were aggregated in the fields of war, in billets behind the lines, in rest camps and at the bases, in hutments and barracks. Those who have ever seen a leave train or a leave boat will know what is meant by overcrowding. Added to this we have to consider that the populations of this and other countries were underfed, and that in addition there was considerable civilian overcrowding. The train, tram and bus services were reduced, and yet more people than ever (and thousands of "leave men" daily) were travelling. Again, money was plentiful, and in consequence the places of amusement were crowded and overcrowded twice or thrice nightly. Probably we are all of us "carriers" of a mild and harmless strain of the influenza germ, and if we imagine that by frequent passage from person to person a harmless germ will become harmful then we have a possible explanation of the influenza epidemic. The poison of influenza had every opportunity of passing rapidly from person to person, and of gaining added virulence at each passage, helped perhaps in turn by the lowered vitality of the people. So much for the theory of the influenza awakening. It is at any rate possible, although it is largely surmise.

Another possibility is that the influenza of 1918-1919 was introduced into Europe from China. Certainly we imported a large number of Chinese as labourers into France in 1917, but on the other hand both we and the French imported people from all the ends of the earth as well as from China to help us in winning the war. Also China, as a source of epidemics, is not altogether popular among epidemiologists. The Black Death of 1346-7 was wrongly attributed to China; nor is it enough in considering the epidemiology of a disease to trace it back to "Cathay, where is the head of the world and the beginning of the earth," and to leave it there without further inquiry—any more than we should track a celebrated murderer to the top of St. Paul's Cathedral and leave him there unregarded. If the influenza really did come from China we must have some more support for this theory.

Indian and Asiatic troops came to this country and to Flanders in the early days of the war. They suffered terribly from respiratory troubles, and they were soon replaced. The Chinese labourers began to arrive in 1916, and there were large numbers in France around Calais and Noyelles in 1917. It is a highly suggestive fact that during the great influenza outbreak of 1918-19 the Chinese, although they had every opportunity of contracting the disease, were relatively immune. This was explained by some as being due to the fact that the Chinese chew garlic, which is a lung antiseptic; but other races and nations—*e.g.*, the Spanish and Italians—eat garlic, and yet suffered from influenza, so this explanation is not acceptable. The Chinese then were not suffering from influenza when they came to France, nor did they appreciably develop it afterwards. It is therefore suggested that the Chinese had a certain immunity to the disease, due perhaps to a previous attack. A curious parallel is found in the history of the first introduction of influenza (the Sweat) into England in the autumn of 1485.

Henry VII., with a rag tag and bobtail crowd of vagabond and rascally soldiery enlisted from around Rouen, landed in England and defeated Richard III. at Bosworth, and then marched to London, where the First English Sweat broke out three weeks later. "There is nothing incredible," says Creighton, "in the supposition that these men had brought a disease into London, although they had not themselves presented the symptoms of that disease. . . . In the case of the singular visitation of England in 1485 the strangers were a swarm of disreputable freebooters from Normandy, natives of a soil that developed the sweat as an indigenous malady in the long course of generations. If they themselves had shown the symptoms of the sweat in 1485 one might have said that the circumstances of their passage in crowded ships and of their exhausting march . . . had brought to the definite issue of a specific disease that which was no more than a habit of body, a constitutional tendency, a disease in the making. But there is no reason to suppose that they themselves incurred the symptoms of the disease at all; it was contact with them in England, particularly in London, that determined the peculiar type of disease in others."

It may be that the explanation of the influenza epidemic in 1918-19 can be found in the consideration of these last two theories rather than in the *de novo* hypothesis; that the Chinese and other foreign workers and troops brought with them new seed of infection into Europe, and that the terrible conditions of those war years provided the manure necessary for fertilising this seed and that these two factors working together produced their harvest of death. This seems to be at least a credible hypothesis. Some recent experimental work by Dr. W. W. C. Topley on epidemics among mice gives support to this explanation, and bears out also Creighton's historical observation regarding the French mercenaries, and their immunity to the sweat. Dr. Topley found that a population of mice which had passed through an epidemic of a disease may remain in apparent good health for considerable periods, but yet be capable of originating a fresh epidemic when mixing with another population which is susceptible to the disease.

---

The Voluntary Hospitals Committee for Surrey, which has now been appointed by the Ministry of Health, will include Lord Cave, Lady Onslow, Lieut-General Sir Edmond Elles, Sir Hedley Le Bas, Mr. R. J. Mellor, Mr. J. Chuter Ede, Alderman G. J. Allen, Mr. F. E. Lemon, Mr. H. A. Powell, Dr. J. Wayte, and Dr. Arnold Lyndon.

---

The effort to build and equip a University for the East Midlands has received an impetus by the munificent offer of Sir Jesse Boot of Highfields Park, a big estate near Nottingham, as a site for the buildings, together with a sum of £100,000 towards the cost of erection. At a meeting of the Governors of Nottingham University College, the Duke of Portland said they hoped soon to lay definite proposals before the Board of Education.

